# An 18-month meditation training selectively improves psychological well-being in older adults: A secondary analysis of a randomised controlled trial

**DOI:** 10.1371/journal.pone.0294753

**Published:** 2023-12-01

**Authors:** Marco Schlosser, Olga M. Klimecki, Fabienne Collette, Julie Gonneaud, Matthias Kliegel, Natalie L. Marchant, Gaël Chételat, Antoine Lutz, for the Medit-Ageing Research Group

**Affiliations:** 1 Division of Psychiatry, Faculty of Brain Sciences, University College London, London, United Kingdom; 2 Department of Psychology, Faculty of Psychology and Educational Sciences, University of Geneva, Geneva, Switzerland; 3 Swiss Center for Affective Sciences, University of Geneva, Geneva, Switzerland; 4 Clinical Psychology and Behavioral Neuroscience, Faculty of Psychology, Technische Universität Dresden, Dresden, Germany; 5 GIGA-CRC In Vivo Imaging, University of Liège, Liège, Belgium; 6 Psychology and Neuroscience of Cognition Research Unit, Faculty of Psychology and Educational Sciences, University of Liège, Liège, Belgium; 7 Normandie Univ, UNICAEN, INSERM, U1237, PhIND "Physiopathology and Imaging of Neurological Disorders", Neuropresage Team, Cyceron, Caen, France; 8 Eduwell Team, Lyon Neuroscience Research Center Inserm U1028, CNRS UMR5292, Lyon 1 University, Lyon, France; Fondazione Policlinico Universitario Agostino Gemelli IRCCS, Universita’ Cattolica del Sacro Cuore, ITALY

## Abstract

**Objectives:**

As the world population is ageing, it is vital to understand how older adults can maintain and deepen their psychological well-being as they are confronted with the unique challenges of ageing in a complex world. Theoretical work has highlighted the promising role of intentional mental training such as meditation practice for enhancing human flourishing. However, meditation-based randomised controlled trials in older adults are lacking. We aimed to investigate the effects of meditation training on psychological well-being in older adults.

**Methods:**

This study presents a secondary analysis of the Age-Well trial (ClinicalTrials.gov: NCT02977819), which randomised 137 healthy older adults (age range: 65 to 84 years) to an 18-month meditation training, an active comparator (English language training), or a passive control. Well-being was measured at baseline, mid-intervention, and 18-month post-randomisation using the Psychological Well-being Scale (PWBS), the World Health Organisation’s Quality of Life (QoL) Assessment psychological subscale, and composite scores reflecting the meditation-based well-being dimensions of awareness, connection, insight, and a global score comprising the average of these meditation-based dimensions.

**Results:**

The 18-month meditation training was superior to English training on changes in the global score (0.54 [95% CI: 0.26, 0.82], p = 0.0002) and the subscales of awareness, connection, insight, and superior to no-intervention only on changes in the global score (0.54 [95% CI: 0.26, 0.82], p = 0.0002) and awareness. Between-group differences in psychological QoL in favour of meditation did not remain significant after adjusting for multiple comparisons. There were no between-group differences in PWBS total score. Within the meditation group, psychological QoL, awareness, insight, and the global score increased significantly from baseline to 18-month post-randomisation.

**Conclusion:**

The longest randomised meditation training conducted to date enhanced a global composite score reflecting the meditation-based well-being dimensions of awareness, connection, and insight in older adults. Future research is needed to delineate the cognitive, affective, and behavioural factors that predict responsiveness to meditation and thus help refine the development of tailored meditation training.

## Introduction

We live in a complex society confronted with unprecedented existential risks and a growing mental health crisis unfolding across generations [1–4]. These complex challenges can disrupt established lifestyles and narratives and expose limitations in both personal and collective capacities for meaning-making [5–7]. The world population is ageing rapidly and older adults present a particularly vulnerable group during these challenging times [8–10]. The physical, social, and psychological difficulties associated with ageing are, today, compounded by the challenges of navigating a fast and uncertain world. Research conducted over the past decades suggested that older adults, despite the physical and cognitive changes associated with ageing, maintain high levels of well-being [11–13]. However, changes over recent years (widespread use of smart phones/internet, COVID-19, geopolitical tensions, increased public awareness of existential risks [e.g., climate change]) might have introduced unique and as-of-yet insufficiently understood pressures on older adults’ psychological well-being (see e.g., [4, 14]). For instance, recent research has indicated that, contrary to expectations expressed during the beginning of the COVID-19 pandemic [4], older adults did not adapt well to the novel psychosocial stressors posed by the COVID-19 pandemic, reporting significant decreases in quality of life, insufficient sleep, and significant increases in the prevalence of depressive and anxiety symptoms [10, 15, 16]. Moreover, the pandemic has led to an increase in the use of at least one psychotropic drug compared to pre-lockdown periods, further highlighting the profound impact of the pandemic on mental health [17]. Understanding how older adults can maintain and deepen their psychological well-being amidst the perils of ageing in today’s complex world presents a pertinent scientific and cultural question.

Over the past decades, research and theory on psychological well-being has aimed to offer answers by understanding the conditions that predict and constitute psychological well-being [18–20]. Psychological well-being is a multidimensional construct. The possibilities and range of human flourishing are deep and wide [21]. Naturally, the conceptions of well-being that have been introduced tended to emphasise different dimensions of human flourishing. In addition to this differentiation, recent theoretical work has increasingly conceptualised psychological well-being as a trainable skill that can be cultivated by specific practices [22]. The cultivation of inner flourishing and the alleviation of suffering have been central tenets of the Greco-Roman philosophical schools as well as Buddhist meditative traditions for millennia but only recently have researchers begun to explicitly synthesise these contemplative perspectives with contemporary scientific models of well-being [22–24].

Particularly Buddhist meditation practices and secular forms of meditation practice derived from Buddhist traditions, including types of mindfulness and loving-kindness and compassion practices, have received a substantial amount of scientific and popular attention [25–30]. Despite this interest and the notable increase in publications on meditation practices, little is known about how these forms of mental training may contribute to human flourishing. Another noteworthy lacuna is the striking lack of research on the effects of meditation training in ageing populations (see [31]). The assumption that well-being is a skill that can be trained not only during periods of seemingly heightened plasticity but across the entire lifespan warrants empirical investigation and suggests that older adults could have the potential to meaningfully enhance their well-being through specific practices [22, 24, 32, 33].

Other important questions of this nascent research field regard the impact of specific meditation practices and the delineation of those well-being dimensions that are particularly amenable to meditation training. Similarly, there is a need for research comparing the utility of meditation training-based theories of human flourishing to prominent scientific models of well-being whose development has not been informed by contemplative perspectives. No line of research or theory can address these complex questions single-handedly. To that end, we employed well-being outcomes that were based on three distinct models of human flourishing: Ryff’s theory of well-being [34], the World Health Organisation’s model of quality of life (QoL; [35]), and the mental training-based framework for well-being developed by Dahl et al. [22].

Ryff [34] proposed a theoretical framework for contemporary scientific perspectives on human flourishing that could unify the largely data-driven and atheoretical research on well-being that had been conducted in this area. Ryff aimed to identify the fundamental dimensions of positive functioning that characterise what it means to be psychologically well. This work proposed six dimensions of well-being, namely self-acceptance, positive relations with others, autonomy (independence), environmental mastery (ability to manage life’s demands), purpose in life, and personal growth (sense of developing and growing; [34]). Ryff’s theory, its accompanying questionnaire (i.e., the 42-item Psychological Well-being Scale; [36], and the vast body of empirical work it generated have significantly shaped the field of well-being research over the past decades [20, 37].

The World Health Organisation (WHO) conceptualises quality of life as an aspect of well-being that reflects “individuals’ perceptions of their position in life in the context of the culture and value systems in which they live and in relation to their goals, expectations, standards and concerns” [35]. Focused on a holistic, cross-cultural approach to health care, the WHO’s work in this area underscores the notion that health is a subjective state of physical and psychological well-being, not simply the absence of disease [35]. The widely used WHO Quality of Life (WHOQOL) assessment includes a psychological domain, which aims to reflect levels of self-esteem, positive feelings (e.g., sense of meaningfulness) and body image, negative feelings (e.g., anxiety), and concentration abilities (i.e., a lack of mind-wandering and distraction, which are associated with lower levels of well-being; see [38]).

Dahl et al.’s [22] mental training-based theory of well-being draws on neuroscientific, psychological, and contemplative perspectives on human flourishing. This model includes a skill-based conception of the well-being dimensions of awareness, connection, and insight. In this framework, awareness can be defined as a heightened, malleable, and undistracted attentiveness to one’s lived experience (including thoughts, feelings, and sensations). Connection encompasses a felt sense of kinship, empathy, gratitude, and understanding toward others that forms the foundation for meaningful interactions and relationships. Insight describes the experiential understanding of how thoughts, feelings, and worldviews contribute to the shaping of one’s perception of self and world [22, 23, 39].

In a recent randomised controlled trial of an 8-week mindfulness-based intervention in older adults at heightened risk of dementia [40], we used outcome measures derived from Ryff’s [34], the WHO’s [35], and three dimensions of Dahl et al.’s [22] models of well-being (i.e., awareness, connection, insight) and found only limited effects. The mindfulness-based intervention was superior to its active comparator (health self-management programme) only on changes in connection at post-intervention, but, in both groups, none of the well-being measures indicated an increase. We concluded that longer intervention studies with waitlist control groups are required to assess if the limited effects could be due to the interventions’ brevity (i.e., 8 weeks) or small base rate changes in well-being in older adults [40]. In contrast to the previous 8-week trial, the present study includes a longer meditation training period (i.e., 18 months), two specific training modules (mindfulness, loving-kindness and compassion), a no-intervention control group, and a different population (i.e., healthy older adults).

We aimed to compare the effects of an 18-month meditation training for older adults on measures of psychological well-being reflective of the three approaches introduced above to a structurally matched English training and a no-intervention condition. These comparisons present a secondary analysis of the Age-Well randomised controlled trial, in which the primary outcomes (mean change in volume and perfusion of the anterior cingulate cortex and insula from pre- to post-intervention) were not significantly modified by the 18-month intervention [41]. Whereas the primary outcome paper only provided data from two time points (i.e., baseline and post-intervention) and compared the meditation group only to the English training group, the present study also presents data from mid-intervention at 9 months, thus offering (i) a more fine-grained analysis of the trajectories of these outcomes in relation to the mindfulness module and loving-kindness and compassion module, (ii) a comparison between the meditation training and no-intervention, and (iii) and a description of the specificity of the newly developed meditation-based composite score (i.e., assessing its trajectory compared those of two established measures of well-being).

In line with prior theory [22, 24], we hypothesised *a priori* that meditation training would cultivate awareness, connection, and insight, and thus, more broadly, also the forms of well-being captured by the PWBS and the WHO’s psychological QoL subscale. We expected these changes in well-being during the meditation training to be superior to English training and no-intervention. We also predicted that during the two-module meditation training, the initial 9-month mindfulness module would primarily train the meditation-based well-being dimensions of awareness and insight, whereas the subsequent 9-month loving kindness and compassion module would primarily cultivate connection. These hypotheses are based on a model of meditation and ageing developed by the European Medit-Ageing Project [24]. In this model, mindfulness practices are hypothesised to enhance psychological well-being by training attentional control, emotion regulation, and meta-cognitive capacities, thereby weakening maladaptive mental schemes and enabling more emotionally balanced states. Compassion practices in this model are hypothesised to enhance well-being by training perspective taking and cognitive reappraisal, fostering caring expressions, perceptions of kinship, and prosocial behaviour. These practices, whether directed towards the self or others, are expected to reduce social stress reactivity through an empathy-based resilience. In this framework, mindfulness and compassion practices have distinct and overlapping mechanisms for improving well-being. Both train meta-awareness and attention control, yet mindfulness practices primarily downregulate maladaptive mental patterns by increasing the malleability, flexibility, and availability of different views and interpretations. Compassion and loving-kindness practices, in contrast, primarily manifest their salutary impact via the upregulation of positive emotions, caring attitudes, and benevolent intentions for self and others. The combination of these practices within a single meditation-based intervention is hypothesised to optimise and synergise the effects of meditation training on older adults [24]. Furthermore, we aimed to assess the potential moderating effects of total amount of practice, responsiveness, expectancy, baseline, sex, cognition, and baseline well-being scores.

## Methods

This study used longitudinal data from the Age-Well randomised controlled trial of the European Union’s Horizon 2020-funded Medit-Ageing European project (public name: Silver Santé Study). The published trial protocol includes detailed information on intervention design, recruitment procedure, eligibility criteria, and assessments [42]. The present study presents a secondary analysis of the Age-Well trial.

### Study design

Age-Well was a monocentric, randomised, controlled superiority clinical trial targeting mental health and well-being in older adults. The three parallel arms comprise a two-module (i.e. mindfulness, and loving-kindness and compassion) 18-month meditation training, a structurally matched 18-month foreign language (English) training, and a passive no-intervention control condition. Participants in the no-intervention group were asked to not engage in meditation or foreign language training during the 18-month period. Randomisation to one of the three groups was performed at a ratio of 1:1:1. Participants were assessed at three visits: pre-intervention at baseline (V1), mid-intervention at 9 months (V2), and post-intervention at 18 months (V3). The primary outcomes of the Age-Well trial, mean change in volume and perfusion of the anterior cingulate cortex and insula from pre- to post-intervention, are reported elsewhere [41].

The intervention was delivered in Caen (France). Written informed consent was obtained from all participants after the procedures had been explained to them and prior to participation. Age-Well received ethics approval from the Comité de Protection des Personnes CPP Nord-Ouest III in Caen (trial registration number: EudraCT: 2016-002441-36; IDRCB: 2016-A01767-44; ClinicalTrials.gov Identifier: NCT02977819). The Age-Well trial has been performed in accordance with the ethical standards laid down in the 1964 Declaration of Helsinki and its later amendments.

### Participants

A total of 137 participants were randomised. Two participants were excluded from the trial after randomisation: one participant presented with amyotrophic lateral sclerosis and one participant had previously experienced a head trauma with loss of consciousness for more than one hour. Another participant died before the end of the trial. The present study thus included data from 134 cognitively unimpaired, older adults (age range: 65 to 78 years [meditation]; 65 to 84 years [English training]; 65 to 75 years [no-intervention]), who had no major neurological or psychiatric disorder, no present or past regular or intensive practice of meditation, were native French speakers, were retired for at least one year, and had completed at least seven years of formal education (Table 1).

**Table 1:** Demographic characteristics.

	Meditation (n = 45)	English training (n = 45)	No intervention (n = 44)
Age, years	69.5 (3.7)	70.3 (4.5)	67.6 (2.5)
Female, n (%)	31 (68.9%)	25 (55.6%)	26 (59.1%)
Education, years	13.1 (3.1)	12.2 (3.0)	14.3 (2.8)

*Note*. All variables are mean (standard deviation) unless otherwise specified.

### Interventions

#### Meditation training

The 18-month meditation training consisted of weekly group sessions (2 hours), daily home practice (≥20 minutes), and one retreat day that involved 5 hours of practice. Informed by existing meditation-based interventions (for details see [42], the meditation training included two 9-month modules that were specifically designed for Age-Well with a focus on developing mindfulness, kindness, and compassion to support healthy ageing and to skilfully meet the physical and psychological challenges associated with ageing. The first 9-month module of the intervention emphasised the training of mindfulness practices, whereas the subsequent 9-month module emphasised the cultivation of loving-kindness and compassion practices.

#### English language training

The English language training followed the same format and structure as the meditation training, and was matched in administration, duration, and dosage of group meetings and home practice. English training (for French native speakers) consisted of exercises aimed at improving participants’ vocabulary and grammatical skills and their application to reading, writing, and speaking. Whereas the meditation training was expected to exert effects on both cognitive control and emotion regulation, the English training was hypothesised to affect cognitive control only.

#### Measures of well-being

The 42-item *Psychological Well-being Scale* (PWBS; [36]) was used to capture psychological well-being. The PWBS is based on a conceptual model of well-being that includes six dimensions: self-acceptance, positive relations with others, autonomy (independence), environmental mastery (ability to manage life’s demands), purpose in life, and personal growth (sense of developing and growing; [34]). A 7-item subscale with a 7-point Likert scale anchored at 1 (strongly agree) and 7 (strongly disagree) is used to measure each dimension. After reverse scoring 21 items, subscale scores were computed by averaging their respective item scores. The total PWBS score was computed by averaging all items. For all scales, higher scores are indicative of higher levels of psychological well-being. Internal consistency of the PWBS subscales has been low to moderate (Cronbach’s alpha ranging from 0.33 to 0.56; [36]).

The *World Health Organization WHOQOL-BREF Quality of Life Assessment* [35] psychological subscale was used to capture psychological quality of life. The WHOQOL-BREF psychological subscale measures levels of positive feelings (e.g., sense of meaningfulness) and body image, self-esteem, the ability to concentrate, and the lack of negative feelings (e.g., anxiety). The 6-item psychological subscale uses a 5-point Likert scale ranging from 0 (not at all) to 5 (completely). After reverse scoring one item, subscale scores were computed by summing the six item scores. Higher subscale scores reflect higher levels of psychological quality of life. The WHOQOL-BREF psychological subscale has displayed good levels of internal consistency (Cronbach’s alpha = 0.81; [35]).

To capture the meditation-based well-being dimensions of awareness, connection, and insight introduced by Dahl et al. [22, 23], we used three previously developed composite scores of meditation-based psychological capacities. Detailed information on the theory-guided development and psychometric properties of these composites have been published [43]. In addition, a global composite score captured the dimensions of awareness, connection, and insight to an equal extent. These composite scores have already been reported as secondary outcomes within the primary outcome paper of the Age-well study [41]. The estimates and p-values presented here slightly differ from the primary outcome paper because the models here used data from all three time points (V1, V2, V3), whereas the primary outcome paper used data from only two time points (V1, V3). Here, we report additional data (i) comparing the trajectories of these composite scores between the mindfulness module and loving-kindness and compassion module (i.e., presenting data from mid-intervention at 9 months), (ii) comparing the meditation training to no-intervention, and (iii) assessing potential moderating effects on the composite scores (see below for a presentation of the potential moderator variables). These additional analyses are exploratory and, therefore, not controlled for multiple comparisons. The three composite scores comprise scales or subscales from six self-report measures (see Table 2), which are described in S1 Table in S1 File.

**Table 2:** Descriptive statistics for well-being outcomes by group and visit based on all available data.

Outcome	Meditation						English training						No intervention					
	Pre		Mid		Post		Pre		Mid		Post		Pre		Mid		Post	
	n	Mean (SD)	n	Mean (SD)	n	Mean (SD)	n	Mean (SD)	n	Mean (SD)	n	Mean (SD)	n	Mean (SD)	n	Mean (SD)	n	Mean (SD)
PWBS																		
Total	45	5.4 (0.6)	45	5.3 (0.7)	45	5.3 (0.7)	45	5.3 (0.7)	45	5.4 (0.7)	45	5.4 (0.7)	44	5.6 (0.6)	44	5.5 (0.6)	44	5.6 (0.6)
Autonomy	45	5.2 (0.8)	45	5.1 (0.7)	45	5.2 (0.7)	45	5.2 (1.0)	45	5.3 (1)	45	5.2 (0.9)	44	5.3 (0.9)	44	5.4 (0.8)	44	5.5 (0.8)
Environmental mastery	45	5.6 (1.0)	45	5.5 (1)	45	5.5 (1.0)	45	5.7 (1.0)	45	5.7 (0.9)	45	5.7 (1.0)	44	5.9 (0.7)	44	5.7 (0.7)	44	5.9 (0.7)
Personal growth	45	5.5 (0.8)	45	5.5 (0.9)	44	5.4 (0.9)	45	5.4 (1.0)	45	5.4 (1)	45	5.1 (0.8)	44	5.7 (0.9)	44	5.7 (0.8)	44	5.5 (0.8)
Positive relations	45	5.7 (0.9)	45	5.5 (1)	45	5.7 (0.9)	45	5.6 (1.0)	45	5.5 (1.1)	45	5.6 (1.0)	44	5.6 (0.9)	44	5.6 (0.9)	44	5.7 (0.9)
Purpose in life	45	5.5 (0.7)	45	5.2 (0.9)	45	5.3 (0.8)	45	5.3 (0.8)	45	5.3 (0.8)	45	5.2 (0.8)	44	5.5 (0.8)	44	5.4 (0.8)	44	5.6 (0.8)
Self-acceptance	45	5.0 (1.0)	45	5 (1)	45	5.0 (1.0)	45	4.9 (1.0)	45	5 (1)	45	5.2 (1.0)	44	5.4 (0.9)	44	5.2 (0.9)	44	5.4 (0.7)
Psychological QoL	45	22.8 (3.1)	-	-	45	23.6 (3.4)	45	24.0 (3.4)	-	-	45	23.6 (3.3)	-	24.1 (3.1)	-	-	44	23.8 (3.0)
Awareness																		
MAIA noticing	45	3.1 (1.2)	45	3.4 (1.1)	45	3.5 (0.9)	45	3.3 (1.1)	45	3.6 (1.1)	45	3.5 (1.0)	44	3.6 (1.1)	44	3.4 (1)	44	3.4 (1.1)
MAIA attention regulation	45	2.7 (1.0)	45	3.1 (0.8)	45	3.4 (0.8)	45	2.8 (0.9)	45	2.9 (0.8)	45	3.0 (0.7)	44	3.0 (0.9)	44	2.9 (0.9)	44	2.9 (0.9)
MAIA emotional awareness	45	3.5 (1.0)	45	3.5 (1)	45	3.8 (0.9)	45	3.5 (1.0)	45	3.5 (0.9)	45	3.6 (0.9)	44	3.4 (1.1)	44	3.6 (1)	44	3.3 (1.0)
MAIA self-regulation	45	3.1 (0.9)	45	3.4 (0.9)	45	3.7 (0.8)	45	3.2 (1.0)	45	3.2 (1)	45	3.4 (0.8)	44	3.0 (1.1)	44	3.3 (1)	44	3.0 (1.0)
MAIA body listening	44	2.3 (1.2)	45	2.8 (1.2)	45	3.2 (1.0)	45	2.7 (1.2)	45	2.7 (1.2)	45	2.7 (1.1)	44	2.5 (1.2)	44	2.7 (1.2)	44	2.5 (1.2)
FFMQ observing	45	9.4 (2.9)	45	9.5 (2.8)	45	9.4 (2.6)	45	8.8 (2.9)	45	8.5 (2.7)	45	9.0 (3.0)	44	10.3 (2.9)	44	9.9 (3.3)	44	9.9 (2.9)
FFMQ act with awareness	45	11.6 (2.1)	45	11 (2.4)	45	11.6 (2.2)	45	11.8 (2.2)	45	11.7 (2.4)	45	11.2 (2.3)	44	11.8 (2.3)	44	12 (2.1)	44	11.8 (2.2)
Connection																		
Compassionate Love Scale	45	92.8 (22.1)	45	95.6 (19.8)	45	98.2 (18.8)	45	88.6 (21.7)	45	85.5 (22.6)	45	85.2 (22.7)	44	90.3 (20.0)	44	90.7 (20.4)	44	89.1 (20)
IRI empathic concern	45	19.4 (4.8)	45	19.4 (4.6)	45	19.4 (4.4)	45	20.2 (4.0)	45	19.6 (3.9)	45	19.1 (4.6)	44	19.9 (4.0)	44	19 (4)	44	18.7 (3.7)
IRI perspective taking	45	17.8 (3.6)	45	17.5 (3.3)	45	17.9 (3.9)	45	16.8 (3.8)	45	16.5 (3.4)	45	16.5 (2.9)	44	17.8 (3.0)	44	17.1 (4.3)	44	17.5 (2.8)
Prosocialness Scale	45	61.8 (7.6)	45	57.3 (9.3)	45	60.6 (9.4)	45	60.1 (7.3)	45	54.7 (10.4)	45	58.2 (10.7)	44	59.1 (9.7)	44	55.7 (10.3)	44	58.3 (7.7)
Insight																		
Drexel Defusion Scale	45	34.4 (5.7)	45	33.5 (6.3)	45	34.6 (6.4)	45	33.8 (5.8)	45	34.1 (6.9)	45	34.0 (7.1)	44	35.1 (5.4)	44	35.4 (7)	44	35.5 (5.7)
FFMQ non-judging	45	11.1 (2.7)	45	11.3 (2.6)	45	11.2 (2.5)	45	11.7 (1.9)	45	11.4 (2.1)	45	11.6 (2.4)	44	12.1 (2.3)	44	12.1 (2.2)	44	12.2 (1.8)
FFMQ non-reactivity	45	9.2 (2.4)	45	9.8 (2.4)	45	10.5 (2.5)	45	9.7 (2.0)	45	9.7 (2)	45	9.3 (2.1)	44	10.3 (2.3)	44	9.9 (2.6)	44	10.0 (2.3)
IRI personal distress^1^	45	18.4 (5.0)	45	18.3 (4.6)	45	18.9 (5.1)	45	17.0 (5.0)	45	17.2 (5.2)	45	17.4 (5.4)	44	18.0 (5.8)	44	18.5 (5.2)	44	18.7 (4.6)

*Note*. PWBS = Psychological Well-being Scale; QoL = Quality of Life; SD = standard deviation; CMBAS = Caring Mindfulness-based Approach for Seniors; HSMP = Health Self-Management Program; PWBS = Psychological Well-being Scale; QoL = quality of life; MAIA = Multidimensional Assessment of Interoceptive Awareness; FFMQ = Five Facet Mindfulness Questionnaire.

^1^Here, higher scores indicate lower levels of distress.

To derive the three composites of meditation-based dimensions of well-being, we first reverse-scored scale scores if lower total scores reflected better functioning so that higher composite scores would indicate higher well-being at all time points. Second, we computed the difference between each scale score at each time point and the baseline pooled mean. Third, we divided these differences by the baseline pooled standard deviation. Fourth, the z-scores of the scales that were assigned to each composite were averaged, yielding three composite scores with a baseline mean of 0 and a standard deviation smaller than one. A global score was computed by averaging the three composite scores. Fifth, to simplify the interpretation of longitudinal data, we re-standardised the composite scores so that within- and between-group changes in the composite scores present changes in standard deviation units.

### Statistical analyses

#### Sample size

Sample size calculations were based on an expected effect size of 0.75 with 80% power and a Bonferroni-corrected two-sided type I error of 1.25% for the two primary outcomes (i.e., the mean change in volume and perfusion of the anterior cingulate cortex from pre- to post-intervention between the meditation and passive control group), resulting in a minimum total number of 126 participants (42 per group), which has been exceeded (n = 137; detailed in [42]).

#### Comparative analyses

To evaluate between-group and within-group differences in mean changes in outcomes, we used one mixed effects linear regression model for each outcome including data from all time points, an interaction term between visit and group, and controlling for baseline scores of the outcome. Positive (negative) estimates of mean between-group differences in changes indicate greater (lower) changes in well-being in the meditation group. Missing data were not replaced and assumed to be missing-at-random. Participant data were not excluded based on very high or low scale scores. Analyses of the well-being outcomes not previously published (i.e., PWBS total scores, psychological QoL) were adjusted for multiple comparison (Bonferroni correction for multiple testing). Exploratory analyses of PWBS subscales and meditation module-specific effects on composite scores (i.e., from V1 to V2 and V2 to V3) were not adjusted for multiple comparison.

To allow for an effect size comparison with previously published meditation-based interventions, we also reported the unstandardised mean change in FFMQ total scores from V1 to V2 and V1 to V3. The FFMQ is a gold standard indicator of the efficacy of meditation-based interventions.

To assess the potential moderating effect on well-being within meditation and English, we used linear regression models with change in well-being scores from V1 to V3 as the outcome and the potential moderator variables as the predictors. Moderator variables included total amount of practice (i.e., combined time spent in class and formal home practice), responsiveness (i.e., combining self-perceived and teacher-rated response to the intervention), expectancy (“How much do you think will the intervention have positively impacted your well-being after 18 months?”), baseline neuroticism (measured by the neuroticism subscale of the 44-item Big Five Inventory [44], sex, cognition (measured by the Preclinical Alzheimer’s Cognitive Composite 5 [45], and baseline scores of the well-being outcome. Additional details of variables included in the moderation analysis can be found in S2 Table in S1 File. R version 4.0.2 and Stata/MP version 16.0 was used for statistical analysis.

## Results

**Fig 1:**
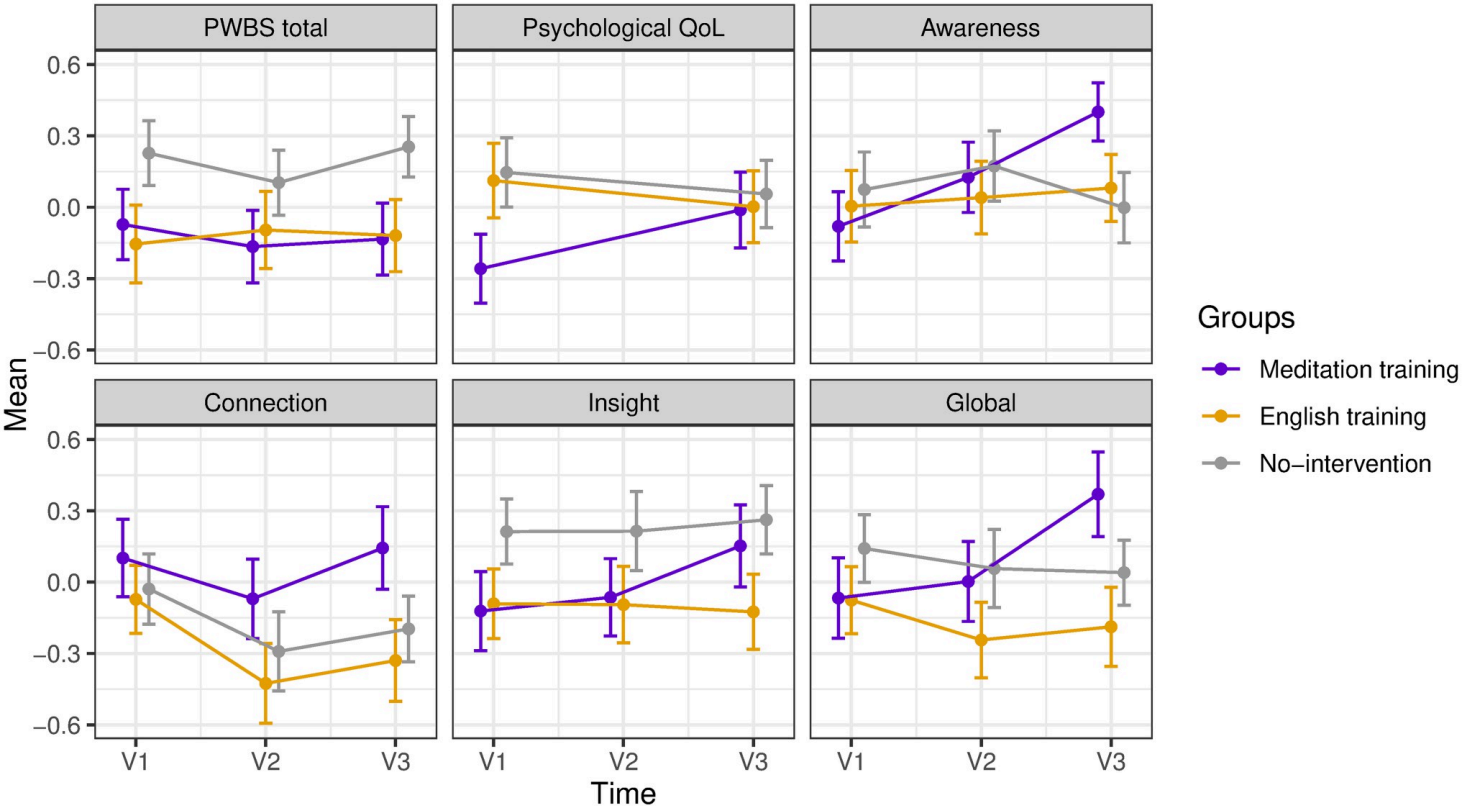
18-month longitudinal changes in Psychological Well-being Scale (PWBS) total scores, and WHOQOL-BREF psychological quality of life (QoL), and meditation-based well-being composite scores (awareness, connection, insight, global) by group. In the meditation group, pre- to mid-intervention (V1 to V2) corresponds to the 9-month mindfulness module, and mid- to post-intervention (V2 to V3) corresponds to the loving kindness and compassion module. The figure displays observed standardised means and SEs (error bars = 1 SE) based on all available data.

Demographic characteristics are displayed in Table 1. Descriptive statistics of well-being outcomes are reported in Table 2 and visualised in Fig 1 (based on all available data). Results from mixed effects regression models evaluating differential change in well-being outcomes are displayed in Table 3 (based on all participants who provided data at V1, V2, and V3).

**Table 3:** Results from mixed effects models assessing differential change in well-being outcomes.

Outcome	Time	Standardised estimated change		No-intervention	Difference in change Meditation vs. English training		Difference in change Meditation vs. No-intervention	
		Meditation	English training		Mean (95% CI)	p	Mean (95% CI)	p
PWBS total^1^	V1 to V3	-0.06 (-0.29, 0.17)	0.04 (-0.20, 0.27)	0.05 (-0.19, 0.28)	-0.10 (-0.37, 0.18)	0.482	-0.11 (-0.38, 0.17)	0.441
Psychological QoL^1^	V1 to V3	0.25 (0.01, 0.48)	-0.11 (-0.35, 0.13)	-0.10 (-0.33, 0.14)	0.36 (0.02, 0.69)	0.037	0.34 (0.01, 0.68)	0.045
Awareness	V1 to V3	0.48 (0.25, 0.72)	0.08 (-0.16, 0.31)	-0.09 (-0.33, 0.15)	0.41 (0.13, 0.69)	0.0045	0.58 (0.29, 0.86)	0.0001
Connection	V1 to V3	0.04 (-0.18, 0.26)	-0.26 (-0.48, -0.04)	-0.18 (-0.40, 0.05)	0.30 (0.04, 0.56)	0.024	0.22 (-0.04, 0.48)	0.101
Insight	V1 to V3	0.27 (0.05, 0.50)	-0.03 (-0.26, 0.20)	0.05 (-0.18, 0.29)	0.31 (0.04, 0.58)	0.026	0.22 (-0.05, 0.49)	0.113
Global^2^	V1 to V3	0.43 (0.19, 0.67)	-0.11 (-0.35, 0.12)	-0.11 (-0.35, 0.13)	0.54 (0.26, 0.82)	0.0002	0.54 (0.26, 0.82)	0.0002

*Note*. Only participants who provided data at all three time points were included in the analyses. All analyses were adjusted for baseline scores of the outcome. CI = confidence interval; PWBS = Psychological Well-being Scale; QoL = quality of life.

^1^These analyses were used a significance threshold of 0.025 adjusted using the Bonferroni correction for multiple testing.

^2^The global composite score reflects the mean score of awareness, connection, and insight.

### PWBS

The differences in the mean PWBS total score changes over 18 months between meditation and English training (Cohen’s d: -0.10 [95% CI: -0.37, 0.18]) or no-intervention (-0.11 [95% CI: -0.38, 0.17]) were not statistically significant (p = 0.48 and p = 0.44, respectively). PWBS total scores did not change during meditation, English training, and no-intervention (Table 3).

Exploratory analyses suggested that across PWBS dimension, PWBS self-acceptance increased over 18 months within English training (0.24 [95% CI: 0.02, 0.47], p = 0.033). No other PWBS dimension changed during meditation, English training, and no-intervention (S3 Table in S1 File).

### Psychological QoL

Differences in the mean psychological QoL changes over 18 months in favour of meditation compared to English training (Cohen’s d: 0.36 [95% CI: 0.03, 0.69], p = 0.037) and no-intervention (0.34 [95% CI: 0.01, 0.68], p = 0.045) were found. However, these differences were not statistically significant after adjusting for multiple comparisons (Bonferroni-corrected significance threshold of p < 0.025). We suggest that the effect sizes of these between-group differences are nonetheless meaningful. Post-hoc analyses indicated that meditation increased psychological QoL (0.25 [95% CI: 0.01, 0.48], p = 0.041), whereas English training (-0.11 [95% CI: -0.35, 0.13], p = 0.362) and no-intervention did not (-0.10 [95% CI: -0.33, 0.14], p = 0.423).

### Meditation-based well-being dimensions

Meditation was superior on changes in awareness to English training (Cohen’s d: 0.41 [95% CI: 0.13, 0.69], p = 0.0045) and no-intervention (0.58 [95% CI: 0.29, 0.86], p = 0.0001). For connection, differences were observed in the mean changes in favour of meditation compared to English training (0.30 [95% CI: 0.04, 0.56], p = 0.024) but not compared to no-intervention (0.22 [95% CI: -0.04, 0.48], p = 0.101). Similarly, for insight, differences were found in the mean changes in favour of meditation compared to English training (0.31 [95% CI: 0.04, 0.58], p = 0.026) but not compared to no-intervention (0.22 [95% CI: -0.05, 0.49], p = 0.113). Meditation was superior on changes in global meditation composite scores to both English training (0.54 [95% CI: 0.26, 0.82], p = 0.0002) and no-intervention (0.54 [95% CI: 0.26, 0.82], p = 0.0002; Table 3).

A frequently used indicator of the efficacy of meditation-based interventions is the mean change in FFMQ total scores. In Age-Well, meditation did not increase FFMQ total scores from V1 to V2 (unstandardised estimate: -0.09 [95% CI: -1.76, 1.94], p = 0.993) or V1 to V3 (1.58 [95% CI: -0.27, 3.43], p = 0.112).

In the meditation group, exploratory analyses assessing the differential effects of the two meditation training modules indicated that the initial 9-month mindfulness module did not significantly increase awareness, connection, insight, or global scores (Table 4, Fig 1), whereas the subsequent 9-month loving kindness and compassion module significantly improved awareness (Cohen’s d: 0.25 [95% CI: 0.01, 0.49], p = 0.034) and global scores (0.38 [95% CI: 0.14, 0.62], p = 0.001). Meditation training was superior to no-intervention only on changes in awareness and global scores during the subsequent 9-month loving kindness and compassion module (i.e., V2 to V3; Table 4).

**Table 4:** Results from exploratory mixed effects models assessing differential change in meditation-based well-being composite scores in the meditation group by training module compared to no-intervention.

Outcome	Time / module^1^	Meditation	No-intervention	Difference in change Meditation vs. No-intervention	
		Mean (95% CI)		Mean (95% CI)	p
Awareness	V1 to V2	0.23 (-0.01, 0.47)	0.08 (-0.15, 0.32)	0.15 (-0.13, 0.43)	0.303
	V2 to V3	**0.25** (0.01, 0.49)	-0.18 (-0.41, 0.06)	**0.43** (0.15, 0.71)	0.003
Connection	V1 to V2	-0.17 (-0.39, 0.05)	**-0.27** (-0.49, -0.05)	0.10 (-0.16, 0.36)	0.455
	V2 to V3	0.21 (-0.01, 0.43)	0.09 (-0.13, 0.32)	0.12 (-0.14, 0.38)	0.370
Insight	V1 to V2	0.06 (-0.17, 0.29)	0.01 (-0.22, 0.24)	0.05 (-0.22, 0.32)	0.713
	V2 to V3	0.22 (-0.01, 0.45)	0.05 (-0.18, 0.28)	0.17 (-0.10, 0.44)	0.224
Global^2^	V1 to V2	0.05 (-0.19, 0.29)	-0.09 (-0.33, 0.14)	0.14 (-0.13, 0.43)	0.305
	V2 to V3	**0.38** (0.14, 0.62)	-0.02 (-0.26, 0.22)	**0.40** (0.12, 0.68)	0.006

*Note*. All analyses were adjusted for baseline scores of the outcome. CI = confidence interval. Estimates in bold were associated p < 0.05.

^1^V1 to V2 corresponds to the 9-month mindfulness module. V2 to V3 corresponds to the 9-month loving kindness and compassion module.

^2^The global composite score reflects the mean score of awareness, connection, and insight.

### Moderator analyses

Exploratory moderator analyses were conducted within meditation and English training groups to evaluate the relationship between baseline characteristics and intervention response over 18 months.

In the meditation group, higher baseline scores of PWBS total, psychological QoL, awareness, insight, and global scores were associated with weaker improvements over 18 months. In the English training group, sex and higher baseline scores of PWBS total, psychological QoL, and awareness were associated with weaker improvements in PWBS total, psychological QoL, and awareness over 18 months. Total amount of practice, responsiveness, and expectancy, neuroticism, and cognition did not consistently moderate the intervention response in either group. Results from the moderator analyses are displayed in S4 Table in S1 File.

## Discussion

The longest randomised meditation training conducted to date enhanced a global composite score reflecting the meditation-based well-being dimensions of awareness, connection, and insight in older adults. We utilised three theory-based conceptions of well-being [22, 34, 35] to test the effects of the longest randomised meditation training to date on psychological well-being in healthy older adults. The 18-month meditation training was superior to English training on changes in awareness, connection, insight, and global scores (comprising awareness, connection, and insight) and superior to no-intervention only on changes in awareness and global scores. Compared to English training and no-intervention, the differences in the mean changes in psychological QoL over 18 months also favoured the meditation training but these between-group differences in change did not remain significant when adjusting for multiple comparisons. There was no evidence for between-group differences in PWBS total score. Post-hoc analyses indicated that within the meditation group, psychological QoL, awareness, insight, and global scores increased significantly over 18 months, whereas none of the well-being outcomes improved within the English training or no-intervention group. Importantly, however, the within-group effect of meditation training on psychological QoL could also have been due to a regression to the mean as raw psychological QoL scores in the meditation group were substantially lower at baseline (and remained lower post-intervention) than those in the English training and no-intervention groups.

Our predictions regarding the differential effects of the 9-month mindfulness module and the subsequent 9-month loving kindness and compassion module meditation-based well-being dimensions (awareness, connection, insight, global) could only be partially confirmed. Exploratory analyses without correction for multiple comparison indicated that the mindfulness module did not significantly increase any of the meditation-based well-being dimensions, although awareness was impacted to a degree that could be deemed meaningful (Cohen’s d = 0.23). One potential explanation is that the 9-month mindfulness module was not long or intense enough to significantly improve meditation-based well-being dimensions in older adults who have never meditated regularly before. The loving-kindness and compassion module, which we expected to exert its most notable effect on connection, significantly increased awareness and global scores while also showing a substantial but non-significant impact on connection and insight (all Cohen’s ds > 0.20). Taken together, in terms of effect sizes, awareness showed a steady increase across both modules, whereas connection, insight, and global scores increased only during the loving-kindness and compassion module. Importantly, our study design does not allow us to conclude that training in loving-kindness and compassion practices is more beneficial for increasing psychological well-being in older adults than mindfulness training, because the prior mindfulness training could have facilitated the impact of the loving-kindness and compassion module. Future dismantling trials with varying trainings are needed to understand potential practice order and interaction effects.

Nonetheless, our results suggest that the duration of meditation training may not be linearly related to improvement in well-being (i.e., a linear dose-response relationship). More frequent sampling of outcome measures of interest during longitudinal studies will help elucidate different trajectories of change for different types of outcomes and meditation practices. Another potential explanation relates to challenging meditation-related experiences that can commonly occur in novice meditators [46–48], but see also [49, 50] and which might have contributed to the unexpected trajectories of connection (i.e., substantial decline) and insight (i.e., no change) during the first nine months of meditation training. Unfortunately, we cannot evaluate to what extent meditation-related difficulties contributed to these counterintuitive results. Although we captured general adverse events in both trials, we did not include a fine-grained, standardised assessment of difficulties that were particularly related to the practice of meditation.

Exploratory moderator analyses indicated that, in line with previous research [40] and theory (e.g., [51]), participants who reported higher levels of psychological well-being at baseline showed a smaller improvement in well-being during the 18-month meditation training (except for connection). Older adults who are psychologically well at baseline seem to benefit less from meditation training than older adults with lower self-rated well-being. This finding, however, might not be specific to meditation training but rather reflective of a general baseline dependence of training outcomes evident in a wide variety of interventions [52]. Future work is needed to also assess the degree to which this pattern reflects potential ceiling effects associated with the measures we employed. The current gold standard measures of well-being were not conceived (and thus might not sufficiently capture) the forms, qualities, and depth of well-being that can potentially be cultivated by long-term meditation training (e.g., meditative absorptions [Pali: jhanas]; see [53–56]).

Notably, higher responsiveness did not consistently predict higher improvements on well-being outcomes during the 18-month meditation training. In other words, those participants whose overall response to the meditation training was perceived by both themselves and their meditation teachers as beneficial did not report greater increases in well-being dimensions than those for whom the impact of the intervention was perceived as less favourable. Furthermore, the total amount formal meditation practice (in class and at home), neuroticism, expectancy, sex, and cognition also did not moderate the effects of the meditation training on well-being measures. In addition, it is worth highlighting that the meditation group consisted of more female participants (68.9%) than the English (55.6%) and no-intervention groups (59.1%). This larger ratio of female participants in the meditation-based group is consistent with our previous 8-week multinational trial (i.e., 64.4%; [40]). Although the literature on differential sex-related responsiveness to meditation training is under-investigated and equivocal [57], some studies indicate that female practitioners show a greater response to meditation training [58]. Although the sex imbalance of the present study could have potentially influenced the effects of the intervention on psychological well-being, our moderator analysis did not support this conclusion. Identifying more cognitive, affective, and behavioural factors that can predict positive responses to meditation training remains an important domain for future research as this line of investigation has the potential to substantially impact the development, refinement, and effectiveness of tailored meditation training.

Two more surprising findings are noteworthy. First, connection actually decreased during the first 9 months of English training and no-intervention. A similar pattern was found in the aforementioned SCD-Well trial [40] in which compassion for others, which was used as a proxy measure for connection, significantly declined within the active comparator group (health self-management programme) over a 6-month period (i.e., from pre-intervention to follow-up). Second, in the present study, 18 months of meditation training had no impact on FFMQ total scores, which, despite its limitations (see [39]), is the current ‘gold standard’ measure of mindfulness and commonly used as a marker of MBIs’ efficacy. In the light of the substantial effects of the 18-month meditation training on other meditation-based dimensions of well-being, this surprising lack of an effect on FFMQ scores further corroborates doubts surrounding the widely used questionnaire’s validity (see [29, 39]).

The Age-Well trial has important strengths. The 18-month meditation training substantially surpasses the shorter-term meditation training periods of previous trials, which have not exceeded several months [26] and represent the largest meditation-based intervention in older adults conducted to date. The meditation intervention followed a manualised training paradigm that was tailored to the needs of older adults and included two training modules to assess the differential effects of specific practices. Acknowledging the strengths and limitations of previous work, we included a theory-based active comparator alongside a no-intervention control, and utilised three distinct theoretical models of well-being to capture diverse dimensions of human flourishing.

The Age-Well trial also has several limitations. Our sample comprised mostly well-educated, healthy participants that were recruited from a single geographic location, whose cultural, economic, and social characteristics may not be representative of other regions, limiting the generalisability of our results to populations of older adults with more demographically diverse compositions. Furthermore, we used previously published composite scores of meditation-based psychological capacities to reflect the well-being dimensions of Dahl et al.’s training-based framework for human flourishing [22]. These self-report scales were designed to capture trait-level individual differences and may be less sensitive to measuring process-level aspects of meditation-based well-being dimensions. Furthermore, the study’s reliance on self-reported data may have introduced a degree of bias, as participants’ responses could have been influenced by their perceptions, memory, or understanding of the questions. Lastly, the present study did not present follow-up data that could assess the trajectory of psychological well-being beyond the intervention period.

Taken together, our findings suggest that meditation training meaningfully impacted select dimensions of psychological well-being (most notably awareness and insight) in healthy older adults, and that these changes were not captured by established standard measures of well-being. Meditation-based interventions could present a promising non-pharmacological approach for the cultivation and enhancement of human flourishing amidst the challenges of ageing in today’s complex world.

### Future directions

We would like to propose several considerations for future work in this area. Beyond the assessment of specific forms of meditation practice and their mechanisms, we recommend assessing meditators’ intentions for practice and other contextual factors [59, 60]. The range of intentions for practice is likely wider than the therapeutic, medical, or even soteriological goals hitherto acknowledged by the science of meditation. Intentions, and a meditator’s relationship to their intentions, might be crucially important for sustaining longer-term meditative training, for deepening meditative skills, and for realising the most desired benefits (e.g., specific states and traits, transformations of perception). In support of this idea, recent evidence showed that combining ethical teachings on virtuous human qualities with mindfulness training led to higher levels of prosocial behaviour than mindfulness training that did not involve such discussions [61]. In general, it will be pertinent for the field to identify and characterise additional factors—including teacher-student relations, the worldviews embedding meditation practices [62, 63], and prior psychedelic experience [64–66]—that could predict and potentially augment the effects of meditation training.

Moreover, another layer of complexity is added to meditation research if we consider that a certain meditation instruction could induce different experiences for different practitioners, or for that matter, the same practitioner at different times. Despite advances in quantifying the effects of meditation practice in psychological and physiological terms, the science of meditation lacks insight into what it is like to meditate. Therefore, whenever possible, we suggest complementing the triangulation aimed at understanding the correlates of meditative states and the effects of meditation training on well-being with first-person phenomenological methods [39, 67–69]. Relatedly, scientific theories of meditation that are informed by nuanced meditation training paradigms (e.g., see [48, 55]) and the lived experience and perceptual skills of long-term meditators will be essential for the maturation of meditation research. It remains to be discussed whether, and to what extent, intensive meditation training for meditation researchers can support this maturation.

### Conclusion

Taken together, this study suggests that longer-term meditation training can enhance important dimensions of psychological well-being in healthy older adults and could thus present a promising non-pharmacological approach for the cultivation of human flourishing. While this finding presents an important contribution to our understanding of the potential of meditation training for enhancing well-being in older adults, there is much more to explore. As we continue this line of research, it will be essential to adopt an interdisciplinary approach that integrates insights from gerontology, psychology, biology (e.g., biomarkers including telomere length), neuroscience, and contemplative studies, and that carefully considers the complexities and nuances of the ageing process. By doing so, future research can further contribute to the development of interventions that support older adults in navigating the challenges of ageing and flourishing in later life.

## Supporting information

S1 File (PDF)
