# Supplementary material for: An 18-month meditation training selectively improves psychological well-being in older adults: A secondary analysis of a randomised controlled trial
Source: PLoS One. 2023 Dec 1;18(12):e0294753. doi: 10.1371/journal.pone.0294753 (PMC10691714; doi:10.1371/journal.pone.0294753)
Supplement: S1 File — (PDF) [file pone.0294753.s001.pdf]

Supporting information

Table S1 Descriptions of the self-report measures included in the meditation-based well-being composites

The *Compassionate Love Scale* (CLS; stranger-humanity version) [1] was used to measure compassion for others. Compassion can be conceptualised as a complex response to suffering – entailing affective, behavioural, and cognitive aspects – that, importantly, includes the intention to reduce suffering. The CLS comprises 21 items with a 7-point Likert scale anchored at 1 (not at all true of me) and 7 (very true of me). Total scales scores are computed by averaging the 21 item scores. Higher total scores reflect higher levels of compassion for others. The CLS has shown high levels of internal consistency (Cronbach's alpha = 0.95) [1]; however, a recent evaluation of the CLS recommended the use of a shorter 7-item version (i.e., COS-7) [2].
The *Drexel Defusion Scale* (DDS) [3] was used to measure levels of defusion, the capacity to psychologically distance oneself from subjective experiences including body sensations, thoughts, emotions and perceptions in general. To be in a state of defusion implies that the seemingly inherent reality commonly assigned to subjective experiences is, to a certain degree, softened, thus making other ways of relating to experience more accessible (e.g. seeing sensations and thoughts as mere phenomenological events or as "just a perception"). The DDS comprises 10 items with a 6-point Likert scale ranging from 0 (not at all) to 5 (very much). Total scores are derived by summing the 10 item scores. Higher total scores reflect a greater ability to defuse from subjective experience. The DDS has displayed good psychometric properties including adequate levels of internal consistency (Cronbach's alpha = 0.95) [3].
The *39-item Five Facet Mindfulness Questionnaire* (FFMQ-39) [4] was used to measure five trait-like facets of mindfulness, namely observing (noticing experiences), describing (labelling experiences), acting with awareness (attending to activities non-mechanically), non-judging (non-evaluative stance towards experiences), and non-reactivity (allowing experiences). The FFMQ-39 comprises one 7-item scale (non-reactivity) and four 8-item scales using a 5-point Likert scale anchored at 1 (never or very rarely true) and 5 (very often or always true). After reverse scoring some items, the subscale scores are derived by summing their respective item scores. Higher subscale scores are indicative of a greater tendency to display the mindfulness facets in daily life. The FFMQ subscales have demonstrated adequate psychometric properties including good internal consistency (Cronbach's alpha ranging from 0.75 to 0.91) [4–6].
The *Multidimensional Assessment of Interoceptive Awareness* (MAIA) [7] questionnaire was used to measure eight state-trait facets of interoceptive awareness, which describe the nervous system's ability to sense, interpret, and integrate signals produced within the body. The 32-item MAIA comprises eight subscales with a 6-point Likert scale anchored at 0 (*never*) and 5 (*always*): noticing (awareness of body sensations; 4 items), not-distracting (not ignoring uncomfortable sensations; 6 items), not-worrying (not distressed by uncomfortable sensations; 5 items), attention regulation (sustaining and controlling attention on sensations; 7 items), emotional awareness (awareness of connection between sensations and emotions; 5 items), self-regulation (regulating distress by attention to sensations; 4 items), body listening (listening to the body for insight; 3 items), and trusting (experiencing the body as safe; 3 items). After reverse scoring some items, subscale scores are computed by averaging their respective item scores. Higher subscale scores are indicative of greater interoceptive awareness accessible to self-report. The MAIA subscales have displayed satisfactory to good levels of internal consistency (Cronbach's alpha ranging from 0.64 to 0.83) [7].

The *Interpersonal Reactivity Index* (IRI) [8] was used to measure empathic tendencies. The IRI comprises four 7-item scales using a 5-point Likert scale ranging from A (does not describe me well) to E (describes me very well). The four scales capture four facets of empathy, namely perspective taking (adopting another's view), empathic concern (feelings of sympathy for others), fantasy (transposing oneself into fictitious characters' experience), and personal distress (feelings of unease in interpersonal dynamics). After converting the letters A-E to 0-4 and reverse scoring some items, scale scores are derived by summing their respective item scores. Higher scale scores reflect higher levels of empathic tendencies and lower personal distress. The IRI scales have shown adequate internal consistency (Cronbach's alpha ranging from 0.75 to 0.82) [8].
The *Prosocialness Scale* [9] was used to measure individual differences in prosocialness including sharing, helping, and taking care of others' needs. The scale comprises 16 items with a 5-point Likert scale anchored at 1 (never/almost never true) and 5 (almost always/always true). Total scores are derived by averaging the 16 item scores. Higher total scores reflect higher levels of prosocialness. The Prosocialness Scale has demonstrated good levels of internal consistency (Cronbach's alpha of 0.91) [9].

Table S2 Details of additional measures included in moderation analysis

Responsiveness<br>We assessed whether and to what degree participants responded to the interventions using data gathered from both participants and teachers. For the meditation training group, a continuous measure of responsiveness was computed by combining standardised scores from two domains: (i) meditation teachers' ratings of participants' response to the intervention and (ii) participants' perceived response to the intervention. Teachers were asked to rate the extent to which they believed each participant benefited from the intervention using a Likert scale ranging from 0 (not at all) to 5 (very much) in addition to rating their perception of participants' levels of connection, positive emotions, negative emotions, and meta-awareness. Participants were asked to rate the levels of connection, positive emotions, negative emotions, and meta-awareness they experienced during the sessions and in daily life. To create the continuous measure of responsiveness for participants in the meditation group, the two teacher-rated and the two participant-rated scores were each standardised and averaged to create a one teacher and one participant score. These two scores, in turn, were then averaged and re-standardised to yield a single responsiveness score with a mean of 0 and a standard deviation of 1. For the English training group, a continuous measure of responsiveness was computed by combining standardised scores from two domains: (i) change from V1 to V3 on an English test and (ii) teacher ratings of participants' response to the intervention. To create a continuous measure of responsiveness for participants in the English training group, both subscores were first standardised using the relevant means and standard deviations. The two standardised domain scores were then averaged and re-standardised create the final responsiveness variable, with a mean of 0 and standard deviation of 1.
Expectancy<br>The question assessing expectancy was adapted from the Credibility Expectancy Questionnaire [10], a self-report six-item questionnaire aimed at assessing intervention credibility and expectancy for improvement. The question measuring expectancy ("A combien pensez-vous que sera l'impact positif sur votre bien-être après l'intervention de 18 mois?"; English translation: "How much do you think will the intervention have positively impacted your well-being after 18 months?") used a Likert scale ranging from 0% (not at all) to 100% (very much).
Cognition as measured by the Preclinical Alzheimer's Cognitive Composite 5 (PACC-5)<br>The PACC-5 is a global cognitive composite used to detect and track cognitive decline related to pre-clinical Alzheimer's disease (AD) [11]. The PACC-5 captures episodic memory, executive function, semantic memory, and global cognition. In Age-Well, the PACC-5 included the Logical Memory test (delayed recall), California Verbal Learning Test (CVLT; delayed free recall), Wechsler Adult Intelligence Scale (WAIS)-IV Coding (raw score), category fluency (total correct) and the Mattis Dementia Rating Scale-2 (total score).

Table S3 Results from exploratory mixed effects models assessing differential change in PWBS dimensions

		Standardised estimated change			Difference in change Meditation vs. English training		Difference in change Meditation vs. No intervention	
Outcome	Time	Meditation	English training	No intervention	Mean (95% CI)	p	Mean (95% CI)	p
Autonomy	V1 to V3	0.04 (-0.22, 0.29)	0.09 (-0.17, 0.34)	0.15 (-0.10, 0.41)	-0.05 (-0.35, 0.25)	0.743	-0.12 (-0.42, 0.18)	0.442
Environmental mastery	V1 to V3	-0.08 (-0.33, 0.16)	0.02 (-0.23, 0.26)	0.09 (-0.16, 0.33)	-0.10 (-0.39, 0.19)	0.489	-0.17 (-0.46, 0.12)	0.241
Personal growth	V1 to V3	-0.10 (-0.38, 0.18)	-0.24 (-0.52, 0.04)	-0.23 (-0.51, 0.04)	0.14 (-0.19, 0.47)	0.404	0.14 (-0.19, 0.46)	0.418
Positive relations	V1 to V3	0.02 (-0.23, 0.27)	0.08 (-0.17, 0.33)	0.09 (-0.17, 0.34)	-0.06 (-0.36, 0.23)	0.678	-0.07 (-0.36, 0.23)	0.664
Self-acceptance	V1 to V3	0.05 (-0.18, 0.28)	**0.24** (0.02, 0.47)	0.05 (-0.17, 0.28)	-0.19 (-0.46, 0.08)	0.159	-0.004 (-0.27, 0.27)	0.977
Purpose in life	V1 to V3	-0.23 (-0.55, 0.09)	-0.07 (-0.39, 0.25)	0.05 (-0.28, 0.37)	-0.16 (-0.54, 0.22)	0.415	-0.28 (-0.66, 0.10)	0.153

Note. Only participants who provided data at all three time points were included in the analyses. All analyses were adjusted for baseline scores of the outcome. Estimates in bold were associated $p < 0.05$. CI = confidence interval; PWBS = Psychological Well-being Scale.

Table S4 Exploratory moderator analyses using linear regression models to predict change in well-being outcomes from V1 to V3

Moderator	PWBS total	Psychological QoL	Awareness	Connection	Insight	Global
**Meditation**						
Practice	0.06 (-0.19, 0.31)	0.04 (-0.23, 0.31)	0.11 (-0.1, 0.33)	0.11 (-0.13, 0.35)	0.02 (-0.22, 0.26)	0.13 (-0.16, 0.42)
Responsiveness	0.2 (-0.09, 0.49)	**0.37** (0.07, 0.66)	**0.24** (0.001, 0.48)	0.02 (-0.24, 0.28)	0.09 (-0.17, 0.35)	0.19 (-0.13, 0.51)
Expectancy	0.04 (-0.22, 0.29)	-0.18 (-0.47, 0.1)	0.04 (-0.18, 0.27)	0.07 (-0.18, 0.32)	-0.03 (-0.29, 0.22)	0.06 (-0.24, 0.36)
Neuroticism at V1	-0.17 (-0.41, 0.07)	-0.23 (-0.48, 0.01)	0.03 (-0.16, 0.22)	-0.09 (-0.3, 0.12)	-0.22 (-0.53, 0.09)	-0.18 (-0.48, 0.12)
Sex (female)	0.03 (-0.47, 0.53)	-0.29 (-0.88, 0.29)	-0.11 (-0.54, 0.31)	0.04 (-0.49, 0.58)	-0.23 (-0.72, 0.25)	-0.01 (-0.6, 0.58)
Cognition	0.15 (-0.2, 0.49)	0.13 (-0.24, 0.5)	-0.02 (-0.32, 0.28)	-0.08 (-0.41, 0.24)	0.17 (-0.17, 0.51)	0.11 (-0.28, 0.51)
Outcome at V1	**-0.42** (-0.71, -0.13)	**-0.5** (-0.8, -0.21)	**-0.59** (-0.81, -0.37)	-0.19 (-0.43, 0.05)	**-0.37** (-0.68, -0.07)	**-0.4** (-0.68, -0.11)
**English training**						
Practice	0.03 (-0.27, 0.32)	0.2 (-0.16, 0.57)	-0.02 (-0.32, 0.29)	0.03 (-0.24, 0.3)	-0.05 (-0.33, 0.24)	-0.05 (-0.37, 0.27)
Responsiveness	0.16 (-0.05, 0.37)	**0.3** (0.03, 0.56)	0.01 (-0.21, 0.23)	0.12 (-0.08, 0.31)	0.18 (-0.05, 0.41)	0.21 (-0.02, 0.44)
Expectancy	0.1 (-0.11, 0.3)	-0.08 (-0.33, 0.18)	-0.04 (-0.25, 0.17)	-0.07 (-0.25, 0.11)	0.01 (-0.2, 0.21)	-0.08 (-0.3, 0.14)
Neuroticism at V1	-0.14 (-0.42, 0.14)	-0.04 (-0.4, 0.31)	-0.07 (-0.31, 0.18)	-0.08 (-0.3, 0.14)	-0.3 (-0.6, 0.01)	-0.19 (-0.46, 0.09)
Sex (female)	**-0.52** (-1.03, -0.01)	**-0.78** (-1.37, -0.2)	**-0.52** (-1.02, -0.02)	-0.25 (-0.68, 0.18)	-0.39 (-0.86, 0.08)	**-0.66** (-1.18, -0.14)
Cognition	0.21 (-0.27, 0.7)	0.32 (-0.29, 0.92)	0.56 (0.05, 1.08)	0.06 (-0.39, 0.51)	0.12 (-0.36, 0.6)	0.44 (-0.1, 0.97)
Outcome at V1	**-0.3** (-0.52, -0.07)	**-0.51** (-0.79, -0.22)	**-0.29** (-0.5, -0.09)	0.02 (-0.18, 0.22)	-0.28 (-0.57, 0.01)	-0.15 (-0.4, 0.1)

Note. All estimates are accompanied by their 95% confidence intervals. Estimates in bold were associated with $p < 0.05$. PWBS = Psychological Well-being Scale; QoL = quality of life.

References (Supporting information)

1. Sprecher, S. & Fehr, B. Compassionate love for close others and humanity. *J. Soc. Pers. Relatsh.* **22**, 629–651 (2005).
2. Schlosser, M., *et* al. The psychometric properties of the compassionate love scale and the validation of the English and German 7-item compassion for others scale (COS-7). *Curr. Psychol.*, 1-13 (2021).
3. Forman, E. M. *et al.* The Drexel defusion scale: A new measure of experiential distancing. *J. Context. Behav. Sci.* **1**, 55–65 (2012).
4. Baer, R. A., Smith, G. T., Hopkins, J., Krietemeyer, J. & Toney, L. Using Self-Report Assessment Methods to Explore Facets of Mindfulness. *Assessment* **13**, 27–45 (2006).
5. Baer, R. A. *et al.* Construct Validity of the Five Facet Mindfulness Questionnaire in Meditating and Nonmeditating Samples. *Assessment* **15**, 329–342 (2008).
6. Gu, J. *et al.* Examining the factor structure of the 39-item and 15-item versions of the Five Facet Mindfulness Questionnaire before and after mindfulness-based cognitive therapy for people with recurrent depression. *Psychol. Assess.* **28**, 791–802 (2016).
7. Mehling, W. E. *et al.* The Multidimensional Assessment of Interoceptive Awareness (MAIA). *PLoS One* **7**, e48230 (2012).
8. Davis, M. H. Measuring individual differences in empathy: Evidence for a multidimensional approach. *J. Pers. Soc. Psychol.* **44**(1), 113–126 (1983).
9. Caprara, G. V., *et al.* A new scale for measuring adults' prosocialness. *Eur J Psychol Assess* **21**(2), 77–89 (2005).
10. Devilly, G. J., *et al.* Psychometric properties of the credibility/expectancy questionnaire. *J Behav Ther Exp Psychiatry* **31**, 73–86 (2000).
11. Papp, K. V., *et al.* Optimizing the preclinical Alzheimer's cognitive composite with semantic processing: The PACC5. *Alzheimers Dement* **3**, 668–77 (2017).
